# Next-Generation Polymyxin Class of Antibiotics: A Ray of Hope Illuminating a Dark Road

**DOI:** 10.3390/antibiotics11121711

**Published:** 2022-11-27

**Authors:** Abdullah Tarık Aslan, Murat Akova, David L. Paterson

**Affiliations:** 1 Department of Internal Medicine, Gölhisar State Hospital, Gölhisar, Burdur 15100, Turkey; 2 UQ Centre for Clinical Research, Faculty of Medicine, University of Queensland, Brisbane, QLD 4029, Australia; 3 Department of Infectious Diseases and Clinical Microbiology, Faculty of Medicine, Hacettepe University, Ankara 06100, Turkey; 4 ADVANCE-ID, Saw Swee Hock School of Public Health, National University of Singapore, Singapore 117549, Singapore

**Keywords:** colistin, SPR206, MRX-8, QPX9003, PBT2, polymyxins

## Abstract

Although new-generation antimicrobials, in particular β-lactam/β-lactamase inhibitors, have largely replaced polymyxins in carbapenem-resistant Gram-negative bacterial infections, polymyxins are still needed for carbapanem-resistant *Acinetobacter baumannii* infections and in settings where novel agents are not readily available. Despite their potent in vitro activity, the clinical utility of polymyxins is significantly limited by their pharmacokinetic properties and nephrotoxicity risk. There is significant interest, therefore, in developing next-generation polymyxins with activity against colistin-resistant strains and lower toxicity than existing polymyxins. In this review, we aim to present the antibacterial activity mechanisms, in vitro and in vivo efficacy data, and toxicity profiles of new-generation polymyxins, including SPR206, MRX-8, and QPX9003, as well as the general characteristics of old polymyxins. Considering the emergence of colistin-resistant strains particularly in endemic regions, the restoration of the antimicrobial activity of polymyxins via PBT2 is also described in this review.

## 1. Introduction

Systemic infections caused by carbapenem-resistant Gram-negative bacteria (CR-GNB) pose high risks of mortality and morbidity and are a public health threat that needs to be urgently addressed [1,2]. In a recent large-scale statistical modeling study, the estimated deaths attributable to bacterial antimicrobial resistance (AMR) in 2019 were reported to be 1.27 million (95% UI 0·911–1·71) worldwide [3]. Third-generation cephalosporin-resistant *Escherichia coli*, carbapenem-resistant *Acinetobacter baumannii* (CRAB), fluoroquinolone-resistant *E. coli*, carbapenem-resistant *Klebsiella pneumoniae*, and third-generation cephalosporin-resistant *K. pneumonia* each caused 50,000–100,000 deaths in 2019 [3]. Given the considerable disease burden and small number of available antimicrobials, the World Health Organization (WHO) declared that CRAB, carbapenem-resistant *Pseudomonas aeruginosa* (CRPA), carbapenem-resistant *Enterobacterales* (CRE), and third-generation cephalosporin-resistant *Enterobacterales* have critical priority for development of novel antimicrobials and future investigations [4].

As a cationic lipopeptide antibiotic, colistin was first discovered as a metabolite of a soil-dwelling bacterium, *Paenibacillus polymyxa* subsp. *Colistinus* [5]. Colistin was first used as an intravenous formulation in the 1950s and was approved by the U.S. Food and Drug Administration (FDA) in 1959 for the treatment of infections caused by Gram-negative bacteria (GNB). However, prescription of colistin largely fell out of favor through the 1970s because of its nephrotoxic and neurotoxic side effects and the availability of new effective and safe antibiotics. In the mid-1990s, due to the emergence of resistant GNB particularly against carbapenems, polymyxins started to be used again for the treatment of infections caused by CR-GNB [5,6]. Despite the excellent bactericidal effect of polymyxins against CR-GNB isolates, clinical efficacy of these antibiotics for CR-GNB infections is not reliable. In parallel with this fact, numerous randomized controlled trials and observational cohort studies have revealed more successful clinical outcomes among patients receiving new-generation antimicrobials than among those being treated with a variety of colistin-containing regimens [7,8,9,10,11]. Moreover, the risk of acute kidney injury is less with novel antimicrobials as compared with colistin-containing combinations [7,10]. Similarly, two randomized controlled trials demonstrated that colistin-containing combination regimens did not confer any benefit over colistin monotherapy in CRAB and CRPA infections [12,13]. For CRE infections, the current literature is somewhat complicated [14,15]. Nevertheless, we may consider using colistin-containing combination regimens only for CRE infections with high INCREMENT scores if new-generation antimicrobials are not readily available [15]. The European Society of Clinical Microbiology and Infectious Diseases (ESCMID) guidelines state that “for patients with severe CRE infections caused by CRE susceptible in vitro only to polymyxins, aminoglycosides, tigecycline or fosfomycin, or in the case of non-availability of new beta-lactam/beta-lactamase inhibitors, we suggest treatment with more than one drug active in vitro” [16].

Despite promising advances in the development of new antimicrobials against CR-GNB infections, the current status is uncertain. Newly approved β-lactam/β-lactamase inhibitors such as ceftazidime-avibactam, imipenem-cilastatin-relebactam, and meropenem-vaborbactam do not have antibacterial activity against metallo-β-lactamase-producing CR-GNB and CRAB strains. Neither relebactam nor vaborbactam inhibit the OXA-48 carbapenemase [15]. The emergence of *Klebsiella pneumoniae* carbapenemase (KPC) variants that confer resistance against ceftazidime-avibactam have been reported even after a relatively short course of antimicrobial therapy [17,18]. Although no single KPC variants have been demonstrated to cause meropenem-vaborbactam resistance until now, reduced expression of OmpK37 porin or OmpK35 and OmpK36 outer membrane porin mutations and/or the overexpression of AcrAB-TolC efflux pump do increase meropenem-vaborbactam MIC values [19,20,21,22]. Likewise, mutations in OmpK35 and OmpK36 porins elevate the MIC values of imipenem-cilastatin-relebactam and some class A GES-type carbapenemase derivatives may confer resistance to this agent [23]. The evolving nature of AMR in GNB requires the development of antibiotics from various classes with different mechanisms of action [24]. Notwithstanding that the size and complexity of existing polymyxins make it difficult, next-generation polymyxins are being developed to reduce toxicity, increase efficacy, and to overcome resistance. [25,26,27]. The discovery and structure–activity relationship studies of novel polymyxin variants have allowed these molecules to enter preclinical investigations, including in vivo toxicity studies [27,28]. Despite significant heterogeneity in the results, several polymyxin molecules with better activity and lower toxicity have been produced, making it highly likely that some of these molecules will reach Phase II/III trials and/or the clinical setting. 

The current manuscript reviews the antibacterial activity mechanisms, in vitro and in vivo efficacy data, and toxicity profiles of next-generation polymyxins that have reached Phase I clinical trials. To achieve the purpose of this article, a thorough literature review was conducted by using Web of Science, Pubmed/Medline, and Google Scholar databases. The key terms included colistin, polymyxin B, colistin resistance, polymyxin resistance, nephrotoxicity, kidney injury, neurotoxicity, polymyxin analogues, polymyxin analogs, new polymyxins, novel polymyxins, polymyxin derivatives, next-generation polymyxins, PMBN, NAB741, SPR741, SPR7061, FADDI, F287, F365, QPX9003, PBT2, MicuRx 12, MicuRx 18, MRX-8, CB-182 804, Pfizer 5x, NAB739, CA824, CA900, VRP-034, Phase I, and Phase I trials. References within the recruited articles were reviewed to capture additional sources. The literature search was undertaken until 1 September 2022 and only articles published in English were evaluated.

## 2. Old Polymyxins (Colistin and Polymyxin B)

### 2.1. General Features

Colistin and polymyxin B have potent in vitro activities against *Enterobacterales*, *P. aeruginosa*, and *A. baumannii*. The activity of colistin and polymyxin B against *Stenotrophomonas maltophilia* exists but is variable [29,30]. For acquired polymyxin resistance mechanisms in CRE, CRAB, and CRPA, detailed information can be found in a previous review [24]. It is important to note that some Gram-negative species have natural resistance against polymyxins, including *Brucella*, *Legionella*, *Campylobacter*, *Vibrio cholera*, *Proteus* spp., *Providencia* spp., some *Aeromonas* spp., *Chromobacterium* spp., *Edwardsiella* spp., *Serratia marcescens*, *Morganella morganii*, *Burkholderia mallei*, and *Burkholderia cepacia*. These bacteria usually have a polymyxin MIC of >32 mg/L, indicating a high level of resistance [31,32,33]. Additionally, polymyxins do not have any anti-Gram positive or anti-anaerobic activity [34].

Polymyxins have a typical structure consisting of ring and tail parts. The ring is composed of a cyclic polycationic peptide and the tail is made up of an acylated tripeptide chain attached with fatty acids at the N-terminus [6]. Colistin and polymyxin B can be distinguished only by the difference in a single amino acid residue in the peptide ring; wherein the D-phenylalanine in polymyxin B is replaced by a D-leucine residue in colistin [35,36]. Colistin is classically administered as a prodrug, namely colistin methanesulfonate (CMS), which is typically given by the intravenous route. The antibacterial mechanism(s) of action of polymyxins is mainly explained by binding of the positively charged polymyxins to the negatively charged phosphate moieties of outer membrane lipids. This interaction disrupts the integrity of the outer membrane and leads to leakage of the cytoplasmic content of GNB [6]. These molecules can also neutralize the activity of the lipid A portion of the lipopolysaccharides which acts as an endotoxin, increase oxidative damage to bacterial DNA and other structural elements via stimulating the production of reactive oxygen species, depolarize bacterial cytoplasmic membrane, and inhibit some essential respiratory chain enzymes of GNB [37,38,39].

### 2.2. Pharmacokinetic/Pharmacodynamic (PK/PD) Properties

The principal PK/PD parameter of polymyxins is the ratio of the area under the concentration time curve for free drug from 0 to 24 h to the minimum inhibitory concentration (MIC) (fAUC_0–24_/MIC) [40,41,42]. Since 20–25% of CMS is transformed into active colistin, >36 h is required to achieve the target serum concentration with widely recommended dosing schedules [43]. This results in a slow increase in free plasma colistin concentration following intravenous (IV) administration of CMS [44,45]. Conversely, colistin can reach high concentrations in urine due to the efficient conversion of CMS into colistin within the urinary tract [46,47]. Therefore, it is recommended to use colistin rather than polymyxin B in urinary tract infections [48]. In a standard patient with 75 kg body weight, a regimen with an intravenous loading dose of 300 mg colistin base activity (9 million IU) and a maintenance dose of 150 mg colistin base activity twice daily is recommended to achieve a plasma steady-state concentration of 2 mg/L [49]. However, PK parameters of colistin are subject to significant interpatient variability, even at a given creatinine clearance [49]. For instance, only <40% of patients with normal renal function can reach >2 mg/L steady-state concentration of colistin, even with a daily dose of 360 mg colistin base activity [49]. Considering equivocal intraepithelial penetration of colistin in the lower respiratory tract, the target serum concentration of colistin (2 mg/L) may achieve the cure of lower respiratory tract infections, if the colistin MIC level of the causative microorganism is lower than 1 mg/L [50]. The daily dose of colistin should be adjusted according to the creatinine clearance of the patients and whether the patients receive hemodialysis support [49]. In dialysis-dependent patients, an additional dose of colistin, corresponding to 10% of the initial dose, should be administered every hour of dialysis to compensate for the loss in dialysis [23].

Polymyxin B exhibits less PK variability due to the fact that it does not need to be converted to its active form after intravenous administration [51]. Furthermore, polymyxin B may be associated with a lower risk of nephrotoxicity than colistin [52]. The maximum concentration of antibiotic in serum (Cmax) can reach approximately 2–14 mg/L after widely administered doses (2.0–2.5 mg/kg loading dose and 1.25–1.5 mg/kg maintenance dose) and its half-life is around 9–11.5 h [53,54,55]. Polymyxin B is eliminated by both renal and non-renal routes. However, its urinary recovery is significantly low (<5%) [56,57,58,59]. It is important to note that polymyxin B has more favorable PK characteristics for infections where it is crucial to attain targeted plasma concentration quickly and reliably. Unlike colistin, the pharmacokinetics of polymyxin B are not significantly altered by kidney function, and therefore, target plasma steady-state concentration of polymyxin B can be attained by approved daily doses, even in those with creatinine clearance levels greater than 80 mL/minute [57,58].

### 2.3. Antibiofilm Activity

Several in vitro and in vivo studies have shown that colistin can have an antibacterial activity on inner layers of biofilm of GNB [60,61,62]. However, the vast majority of these studies have tested high concentrations of colistin (10–25 mg/L), which are difficult to reach in a biofilm-associated infection. To circumvent this issue, high-dose polymyxin regimens can be combined with other antimicrobial agents. In vitro and in vivo studies have both indicated favorable activity with colistin in combination with other agent(s) as each agent has different targets within the biofilm structure [60,61,62]. Clinically, biofilm growth may occur in device-related infections, prosthetic joint infections, and lower respiratory tract infections of cystic fibrosis patients. To date, there is little clinical experience with the use of polymyxins in these settings. Nonetheless, to attain higher local concentrations at the site of the infection, colistin can be administered locally via aerosolized, intraventricular routes, and cement spacers. Although the comparative efficacy between colistin alone or in combination have not been gauged in these infections, local administration of colistin for cystic fibrosis, non-cystic fibrosis bronchiectasis, prosthetic joint infection, and central nervous system device-related infections may confer beneficial clinical outcomes [63,64].

### 2.4. Toxicity

Polymyxins have notoriously neurotoxic and nephrotoxic side effects in a dose-dependent manner. Fortunately, both side effects are generally reversible after discontinuation of the offending drug [65]. Polymyxins-associated neurotoxicity can present with paresthesia, weakness, visual disturbances, dizziness/vertigo, ataxia, confusion, neuromuscular blockade, and apnea [66]. The most common neurologic side effect is paresthesia; the rarest are apnea and neuromuscular blockade [66]. The nephrotoxicity risk is substantially increased when the plasma concentration of colistin exceeds 2.5 mg/L, and colistin-associated acute kidney injury is reported to be seen in up to one-half of colistin-receiving patients [67,68]. Polymyxin-associated kidney injury significantly correlates with underlying renal dysfunction, older age, concomitant nephrotoxin exposure, and duration of therapy [69,70,71]. Polymyxins’ nephrotoxic effect is primarily mediated by increased oxidative stress, mitochondrial damage, and impaired tubular epithelial permeability [65]. 

## 3. Why Do We Need to Develop Next-Generation Polymyxins?

Clinicians should be extremely careful while assessing the results of studies comparing the efficacy of polymyxins with other agents for the treatment of CR-GNB infections. Some important factors, including existence of various treatment regimens in the comparison groups, high frequencies of combination regimens used in both the polymyxin arm and the comparator arms, and the suboptimal dosing of polymyxins in many studies preclude clinicians from drawing strong conclusions. While one review has concluded that the majority of polymyxin-induced kidney injury is mild and reversible, and does not result in a higher mortality rate or the need for renal replacement therapy [72], other studies have shown almost one-third to one-half of patients treated with colistin-containing regimens for CR-GNB infections developed acute kidney injury, and up to two-thirds of these patients had 30-day or in-hospital mortality [72,73,74,75,76,77,78,79]. Similarly, poor clinical outcomes (e.g., high clinical failure and prolonged hospital stay) have been documented with colistin-based regimens for treating CR-GNB infections [80,81,82,83]. Given suboptimal PK/PD indexes, particularly in lung, bone, and the central nervous system, and limited efficacy and increased risk of toxicity pertaining to colistin use, the Clinical and Laboratory Standards Institute (CLSI) has recently modified breakpoints of polymyxins for *Enterobacterales*, *P. aeruginosa*, and *Acinetobacter* spp. CLSI removed the susceptibility category of polymyxins and the ‘intermediate’ breakpoint for these bacteria was established at ≤2 mg/L, suggesting unpredictable clinical efficacy of polymyxins even for GNB with a MIC level of 2 mg/L [84]. Similarly, in 2022, the European Committee on Antimicrobial Susceptibility Testing (EUCAST) issued a warning promoting the use of colistin as a combination therapy for systemic CR-GNB infections other than urinary tract infection. Therefore, the susceptibility breakpoints of colistin were shown in brackets for *Enterobacterales*, *Pseudomonas* spp., and *Acinetobacter* spp. as follows: (2), (4), and (2) mg/L [85]. Some patient-related factors can also limit colistin use in critically ill patients, including augmented renal clearance, obesity, proclivity to development of colistin-associated side effects, and increased volume of distribution [86]. The daily use of polymyxins is further complicated by the failure of routine susceptibility tests to detect colistin susceptibility among GNB. These tests (e.g., disk diffusion testing and automated systems) may falsely detect a significant fraction of CR-GNB isolates as susceptible, while in fact, they are non-susceptible according to the standard broth microdilution method [87]. Lastly, irrational utilization of polymyxins, not only in human medicine, but also in veterinary medicine, has dramatically increased the frequency of polymyxin-resistant Gram-negative microorganisms in endemic regions [71,88,89]. In this dreadful scenario, next-generation polymyxins are more than welcome for the expanding antibiotic pipeline against CR-GNB infections. Even though new antimicrobials for the treatment of CR-GNB infections have been introduced to the market during the last decade, there is not yet a ‘perfect’ molecule that can inactivate all types of CR-GNB and fully meet the needs of every patient. In particular, new agents with a spectrum of activity against CRAB and metallo-β-lactamase-producing GNB are still in great demand, and new polymyxins may, therefore, be an option for the treatment of these infections.

## 4. Novel Polymyxin Molecules

Over the last 5–10 years, a significant amount of public money has been granted for programs that purpose to design new polymyxin variants. The U.S. National Institute of Allergy and Infectious Diseases (NIAID) has supported Prof. Roger Nation and Prof. Jian Li at the Monash University (Melbourne, Australia) for their polymyxin programs. Likewise, NIAID has funded Spero Therapeutics (Cambridge, MA, USA) for preclinical studies on SPR206 and CARB-X has supported MicuRx Pharmaceuticals, Inc. (Hayward, CA, USA) and Spero Therapeutics for further development of MRX-8 and SPR741, respectively.

Next-generation polymyxins do have important potential for progressing through the preclinical and clinical phases and might confer major advantages over old-fashioned polymyxins. However, it should not be forgotten that many difficulties and unsuccessful attempts have been encountered during this process. Due to toxicity issues, Cubist halted progress in the development of CB-182 804 after Phase I clinical studies. Later, Pfizer discovered a new polymyxin analogue containing diaminopropionic acid (Dap) instead of diaminobutyric acid (Dab) at the R3 position in the polymyxin peptide. Although this molecule reduced the toxicity by well over two-fold in kidney cell cultures and rat kidneys, these findings could not be shown in dogs [27,90]. AstraZeneca stopped the polymyxin program even before revealing the chemical structures of their new polymyxin molecules. Despite promising in vitro results, Spero Therapeutics did not proceed from Phase I to Phase II studies for SPR741. Since SPR741 has only permeabilizing effect without any direct antibacterial activity, and SPR206 with permeabilizing effect and direct antibacterial activity is being developed by the same company, clinical development of SPR741 has been halted by the manufacturing company. For antimicrobial compounds that have completed all preclinical studies, it is operationally difficult, expensive, and time-consuming to successfully progress in clinical trials (Phases I–III) against carbapenem-resistant organisms and to obtain regulatory approval. For example, only 16.3% of antibacterial therapeutics reaching Phase I trials have been approved by the U.S. FDA in the last decade. To this end, in addition to some polymyxin analogues that have reached and/or completed Phase I clinical trials, such as SPR206, MRX-8, and QPX9003, PBT2 is discussed in detail later in this article. 

### 4.1. SPR206

SPR206 has a modified fatty acyl tail with an aryl chloride group substituted aminobutyryl N-termini and a shortened nanopeptide cyclic core with L-Dap residues attached to the peptide ring. In an outer membrane interaction kinetics study, SPR206 showed almost the same outer membrane lipopolysaccharide binding affinities as polymyxin B, but higher affinities than other SPR analogues (e.g., SPR1205 and SPR946). Furthermore, SPR206 was found to be significantly more effective than SPR741 in permeabilization experiments, indicating that it might be highly suitable for employment in combination therapy which is generally preferred in serious infections [91].

In a study by Zhang et al., SPR206 displayed excellent in vitro activity against colistin-susceptible carbapenem-resistant OXA-harboring *A. baumannii* (MIC_50/90_ = 0.064/0.125 mg/L), KPC-2-producing *Enterobacterales* (MIC_50/90_ = 0.125/0.5 mg/L), and NDM-expressing *Enterobacterales* (MIC50/90 = 0.125/0.25 mg/L) [92]. Indeed, SPR206 was the most potent antimicrobial studied, with from two- to four-fold lower MICs than old-fashioned polymyxins for *A. baumannii*, *P. aeruginosa*, and *Enterobacterales*. Similarly, MIC levels of SPR206 (MIC_50/90_ = 0.064/0.125 mg/L) were significantly lower than those of tigecycline (MIC_50/90_ = 2/2 mg/L) for tigecycline-susceptible CRAB [92]. These results were confirmed by two recent studies that showed potent activity of SPR206 against contemporary collections of *A. baumannii*, *P. aeruginosa*, and *Enterobacterales*, including multidrug-resistant and carbapenem-resistant isolates from the USA [93,94]. Despite these promising results, SPR206 had only comparable antibacterial activity to old polymyxins against colistin-resistant strains [92]. It should be highlighted that the in vitro susceptibility studies comparing new polymyxin derivatives with old-fashioned polymyxins are more difficult to perform than classical susceptibility studies. The results of these studies can be affected by several factors such as the selected growth medium and adsorption of polymyxins to polystyrene microwell plates and other plastic materials, which is dependent on their hydrophobicity and cationic charge [25,95]. To overcome these issues, while performing the broth microdilution method, cation-adjusted Mueller–Hinton broth (CAMHB) should be preferred as a growth medium. Additionally, to minimize the effect of adsorption, polymyxins should be added to the wells that already have the target bacteria in the growth medium. 

The dose–response relationships for SPR206 and polymyxin B were nearly identical in a neutropenic mouse thigh infection model with polymyxin B-susceptible *A. baumannii* [96]. In a neutropenic mouse lung infection model targeting the same *A. baumannii* strain, the efficacy of SPR206 administered at various doses (2.15, 8.6, 17.2, and 25.8 mg/kg/dose) was compared with that of polymyxin B administered at 17.2 mg/kg/dose [95]. Intriguingly, despite polymyxin B not showing any effect on the development of the infection, the same dose of SPR206 mitigated the bacterial burden in the lungs by 1.6 log_10_ CFU/g as compared with the initial bacterial load. When SPR206 was dosed at 25.8 mg/kg/dose, it was still well tolerated and able to reduce bacterial burden by 3.2 log_10_ CFU/g as compared with the pretreatment level. The poor antibacterial activity of polymyxin B in neutropenic mouse lung infection models has previously been reported and can be explained by inefficient penetration of polymyxin B in epithelial lining fluid [97,98]. Brown et al. also performed an in vivo mice nephrotoxicity model which included assessment of renal injury biomarkers and histopathological examinations after exposure to SPR206 [96]. The renal injury biomarkers were significantly higher in urine after polymyxin B exposure as compared with SPR206. Consistently, histopathological examinations have shown no signs of renal parenchymal injury in SPR206 receiving mice even after exposure to 25 mg/kg/dose [96]. Other animal nephrotoxicity models using mice and monkeys have also demonstrated a reduced risk of nephrotoxicity with SPR206 as compared with polymyxin B [99]. SPR206 also exhibited great efficacy in murine thigh and lung infection models using multidrug-resistant *P. aeruginosa* and *A. Baumannii* [100]. These studies demonstrated that SPR206 was twice as effective as the maximum tolerated dose of polymyxin B in a murine lung infection model and it reduced *P. aeruginosa* bacterial load by 3.6 log_10_ CFU/g, 24 h after its subcutaneous administration. Superior antimicrobial activity of SPR206 may come from the aryl chloride group at the N-terminus, which has been shown to contribute to the antimicrobial activity of vancomycin in previous studies [101].

In a Phase I clinical study involving 94 healthy adult volunteers, SPR206 was found to be generally well tolerated, following 1 h of IV infusion at single doses from 10 mg to 400 mg and multiple doses from 25 mg to 150 mg every 8 h for 7 days and 100 mg every 8 h for 14 days [102]. The adverse events were mostly mild in severity and dose-dependent. In addition, systemic exposure to SPR206 was dose proportional, with time to peak between 1.1 and 1.3 h. The plasma half-life ranged from 2.4 to 4.1 h and steady-state concentration was attained by Day 2. The percentage of the dose excreted in the urine as unchanged SPR206 may exceed 50% in a dose-proportional manner. Importantly, acute kidney injury was not observed over the 14 days of 100 mg every 8 h dosing of SPR206, which is a dosing regimen expected to be used in Phase II and Phase III clinical trials. Although the results of the Phase I study investigating the pharmacokinetics of SPR206 (NCT04865393) in patients with varying degrees of renal function have not yet been reported, the results of another Phase I study exploring the intrapulmonary PK characteristics of SPR206 (NCT04868292) were presented in the ID Week 2022. Given the importance of antibiotic concentrations in epithelial lining fluid and alveolar macrophages for determining the activity and dosing of antibiotics in lower respiratory tract infections, Rodvold et al. demonstrated that the ratios of AUC_0–8_ in epithelial lining fluid and alveolar macrophages to plasma free SPR206 were 0.264 and 0.328 in healthy volunteers, respectively. Additionally, exposure of three doses of SPR206 100 mg IV every 8 h was well tolerated [103]. 

Spero Therapeutics announced that a Phase II, cross-indication resistant pathogen clinical trial designed to enroll patients with complicated urinary tract infection, hospital-acquired and ventilator-associated bacterial pneumonia, and bloodstream infections is assumed to be initiated in the third quarter of 2023.

### 4.2. QPX9003

Although development of more potent and safer polymyxin variants is very challenging owing to the complex interactions between structure and efficacy, nephrotoxicity, epithelial lining fluid concentration, and lung surfactant binding, alterations in various non-conserved positions (e.g., positions 3, 6, 7, and N-terminal fatty acyl group) within the polymyxin scaffold have led to the generation of a promising synthetic lipopeptide, which has less nephrotoxicity risk, a wider therapeutic window, better systemic drug exposure, and higher efficacy in pulmonary infections as compared with old polymyxins [104].

A new lipopeptide polymyxin B variant called QPX9003 (formerly F365) was discovered by Jian Li and his colleagues at Monash University (Australia) and developed by Qpex Biopharma Inc. (San Diego, CA, USA). This compound was selected as a lead candidate to progress forward from various synthetic lipopeptide polymyxin derivatives (e.g., F287) due to its excellent in vitro potency against target pathogens and safer nephrotoxicity profile. The chemical structure of polymyxin B was substituted by 2,4-dichlorobenzoyl at the N-terminus, as well as Dap, D-leucine, and L-2-aminobutyric acid at positions 3, 6, and 7, respectively [105]. Although the hydrophobicity is reduced at both the N-terminus and positions 6 and 7 as compared with polymyxin B, QPX9003 seems to retain the capacity to form a folded conformation comparable to polymyxin B upon binding with outer membrane lipopolysaccharides [104]. Furthermore, replacement of the Dab residue with Dap at position 3 does not change the electrostatic interactions of position 3 with the ketodeoxyoctonic acid moiety [104]. Due to these optimizing modifications, QPX9003 has been reported to confer slightly better in vitro potency against *P. aeruginosa*, *A. baumannii*, and *K. pneumoniae* isolates and has not been associated with nephrotoxicity, even at a dose up to 72 mg/kg/d in a mouse model [104]. In these experiments, the MIC_50_/MIC_90_ values were 0.5/1 μg/mL, which were two-fold lower than those of polymyxin B (MIC_50_/MIC_90_, 1/2 μg/mL) against CRPA (n = 213) isolates. Likewise, QPX9003 (MIC_50_/MIC_90_, 0.25/1 μg/mL) was up to four-fold more potent than polymyxin B (MIC_50_/MIC_90_, 1/4 μg/mL) in susceptibility assays of CRAB strains (n = 210). The in vitro susceptibility results of the same pathogens were also analyzed in the presence of a natural bovine lung surfactant extract. The MICs of old polymyxins were elevated almost eight-fold, whereas the MICs of QPX9003 were not affected at all in these analyses. In addition, QPX9003 was remarkably less prone to the development of resistance than polymyxin B in serial passaging experiments [104]. In experiments confirming the safer properties of this molecule, no nephrotoxic effect was observed after intraperitoneal administration of 150 mg/kg of QPX9003 [104]. In parallel with this fact, renal histopathological examinations demonstrated mild-moderate tubular degeneration at the highest examined dose of 50 mg/kg/day QPX9003 in 12.5–25% of cynomolgus monkeys [104].

QPX9003 was also tested in a neutropenic mouse lung infection model against polymyxin-susceptible multidrug-resistant clinical isolates of *K. pneumoniae*, *P. aeruginosa*, and *A. baumannii*, including carbapenem-resistant strains. At the highest intrapulmonary dose that could be safely administered for polymyxin B (45 mg/kg/day), both polymyxin B and QPX9003 did not display any significant antimicrobial activity at 24 h against these clinical strains. Contrary to these results, considering its safer nephrotoxicity profile, when the intrapulmonary dose of 90 mg/kg/day QPX9003 was tested in mouse pneumonia models with the same bacterial isolates, QPX9003 was found to have significant killing effects against all bacteria [104]. In a neutropenic mouse thigh infection model, QPX9003 exhibited >2.0 log_10_ reduction in CFU/thigh as compared with polymyxin B against a polymyxin-susceptible CRAB clinical isolate. Intriguingly, in neutropenic mouse pneumonia models using polymyxin-resistant *A. baumannii*, *P. aeruginosa*, and *K. pneumoniae*, QPX9003 showed significant bactericidal effects against polymyxin-resistant strains, with reductions in bacterial loads up to 4.65 log_10_ CFU/lung. These findings underlined the importance of a wider therapeutic window and less propensity to binding to lung surfactant for reliable pulmonary drug exposure [104].

Plasma protein binding of QPX9003 has been reported to be significantly lower than that of polymyxin B [104]. Although it had a shorter half-life than polymyxin B, QPX9003 had a higher plasma fAUC than polymyxin B in mice and rats. The urinary recovery of QPX9003 was four-fold higher than polymyxin B in rat models, possibly due to reduced reabsorption of QPX9003 by the kidneys. In PK analyses of mouse pulmonary epithelial lining fluid, QPX9003 attained a three-fold higher Cmax than polymyxin B after a single subcutaneous dose of 40 mg/kg. Although the epithelial lining fluid AUC level of QPX9003 (86.2 mg·h/L) was similar to that of polymyxin B (80.0 mg·h/L), because of the remarkable loss of antibacterial activity of polymyxin B due to binding to lung surfactant, the expected fAUC level of QPX9003 in epithelial lining fluid would be almost eight-fold higher than that for polymyxin B. Together, QPX9003 has in vitro activity comparable to colistin against colistin-resistant CR-GNB. However, due to its better PK properties, a significant killing effect was demonstrated in the mouse lung infection model, including against GNB isolates with polymyxin B MICs of 4–8 mg/L.

A Phase I clinical trial of QPX9003 enrolling 104 healthy adult subjects (NCT04808414) was commenced on 3 June 2021 and completed on 14 July 2022. Even though the results of this trial have not yet been published in an academic journal, the preliminary results were presented in the ID Week 2022 [105]. In this presentation, QPX9003 was reported to be safe and well tolerated at all doses tested. Its plasma AUC_0–∞_ (2.7–108.3 mg.h/L) and Cmax (0.8–24.3 mg/L) increased with increasing doses with a mean half-life from 2.6 to 4.4 h. Based on these data, further clinical development of this molecule is warranted. 

### 4.3. MRX-8

MRX-8 is a next-generation polymyxin that carries a fatty acyl tail linked via an ester bond that is being developed by Shanghai based MicuRx. After administration of MRX-8, cleavage of this ester bond leads to the formation of a less toxic metabolite without any loss of antimicrobial activity. Unlike polymyxin B, MRX-8 was developed using a new method called “soft drug design”, which has typically been used to create new drugs with reduced toxicity and increased therapeutic index by integrating metabolism and detoxification factors into the drug development process [106,107,108].

An in vitro study evaluated the activity of MRX-8 against *Enterobacterales*, *P. aeruginosa*, *A. baumannii*, *S. maltophilia*, *B. cepacia*, *Alcaligenes* spp., and *Haemophilus* spp. clinical isolates (n = 765) collected from 2017 to 2020 in China [106]. The MIC_50/90_ of MRX-8 was 0.125/0.25 mg/L and 0.06/0.125 mg/L for carbapenem-susceptible (n = 58) and carbapenem-resistant *E. coli* strains (n = 46), respectively. It was 0.25/0.5 mg/L for carbapenem-susceptible *K. pneumoniae* strains (n = 46) and 0.125/0.5 mg/L for carbapenem-resistant *K. pneumoniae* isolates (n = 60). Similar to other polymyxin derivatives, MRX-8 had no more superior antimicrobial activity than that of old-fashioned polymyxins for polymyxin-resistant *E. coli* (n = 18) and *K. pneumoniae* (n = 32) isolates (MIC_50/90_ of MRX-8 was 4–16/>32 mg/L). For carbapenem-susceptible *P. aeruginosa* (n = 46) and CRPA strains (n = 54), the MIC_50/90_ values of MRX-8 were 1 mg/L. They were 0.5 mg/L for carbapenem-susceptible *A. baumannii* isolates (n = 43) and 0.5/1 mg/L for CRAB strains (n = 70). Lastly, for naturally polymyxin-resistant strains, including *Providencia* spp., *Proteus* spp., *Serratia* spp., *Morganella* spp., *S. maltophilia*, and *B. cepacia*, the MIC_50_ values of MRX-8 were >32 mg/L.

The pharmacodynamics analysis of MRX-8 and polymyxin B against a variety of GNB in murine thigh and lung infection models have revealed that MRX-8 and polymyxin B both had similar AUC/time from 0 h to infinity exposures (0.22 to 12.64 mg · h/liter vs 0.12 to 13.22 mg · h/liter) [108]. There were linear Cmax and AUC_0–∞_ values over the dose range for both agents. Dose fractionation experiments demonstrated that the Cmax/MIC and AUC/MIC ratios of MRX-8 both had robust associations with antimicrobial efficacy. In the thigh infection models with *E. coli*, *P. aeruginosa*, *K. pneumoniae*, and *A. baumannii*, both agents exhibited better antimicrobial efficacy with increasing doses. Even though AUC/MIC ratios ensuring net stasis for *E. coli* and *K. pneumoniae* were similar for MRX-8 and polymyxin B, this ratio was numerically smaller for MRX-8 than for polymyxin B in murine thigh infection models utilizing *P. aeruginosa* and *A. baumannii* strains [108]. Nevertheless, considering the small number of pathogens tested in these experiments, this result should be evaluated cautiously. In the lung infection model, MRX-8 was found to have more favorable in vivo activity than polymyxin B at similar systemic antibiotic exposures being measured by free-drug AUC values [108].

**Figure 1:**
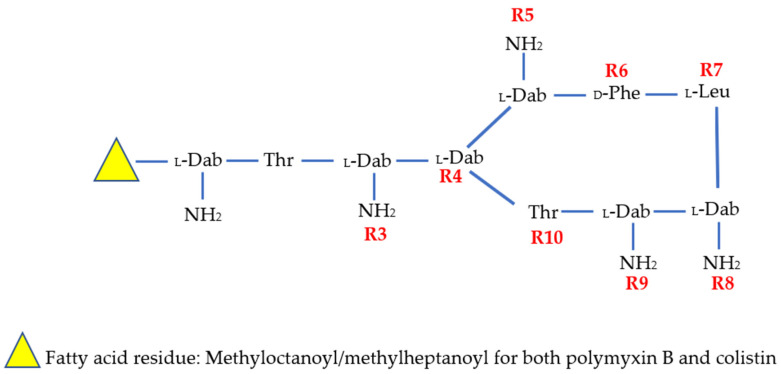
Chemical structure of polymyxin B and colistin. Amino acid residue differing between polymyxin B and colistin at R6 position: _D_-Phe for polymyxin B and _D_-Leu for colistin. Abbreviations: Dab, diaminobutyric acid; Thr, threonine; Phe, phenylalanine; Leu, leucine.

**Figure 2:**
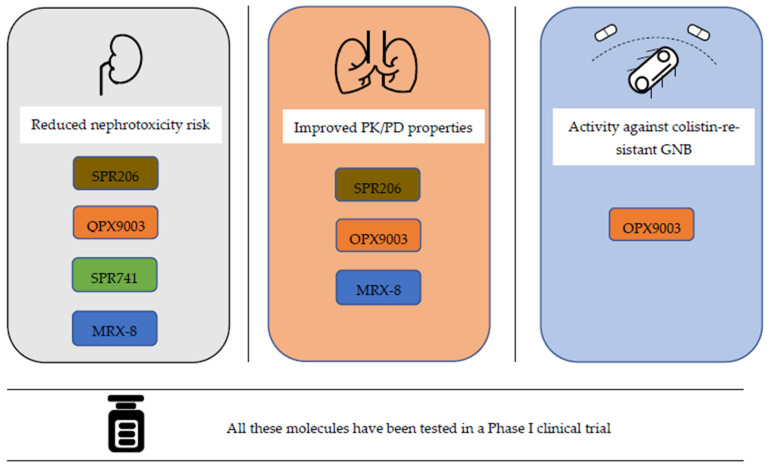
New polymyxin variants were developed primarily for three main purposes. The first is to reduce the risk of nephrotoxicity. The second is to improve PK/PD properties, thereby, increasing clinical efficacy in lower respiratory tract infections. The third is to ensure antimicrobial activity against pathogens resistant to old polymyxins. This figure presents the distribution of next-generation polymyxin derivatives that have reached and/or completed Phase I clinical trials and met these predetermined goals.

As the Wellcome Trust-funded Phase I trial of MRX-8 is currently ongoing (NCT04649541), PK and safety data from a human trial of MRX-8 are not yet available. The chemical structures of new polymyxin variants are depicted in Table 1 and Figure 1. Additionally, Figure 2 classifies new polymyxins according to the predetermined main objectives of their development.

## 5. PBT2

Alternative antimicrobial agents are urgently needed for WHO’s top priority Gram-negative pathogens. In addition to de novo synthesis of new molecules, repurposing drugs constitutes a viable alternative, which is time and cost effective. A novel compound-2-(dimethylamino) methyl-5,7-dichloro-8-hydroxyquinoline-hydroxyquinoline analog ionophore has been shown to restore the antimicrobial activity of polymyxins against polymyxin-resistant GNB regardless of the lipopolysaccharide modification mechanisms associated with polymyxin resistance. This orally bioavailable compound can mediate metal ion transfer (e.g., Zinc) across cellular membranes. The principal mode of action for PBT2 is disruption of metal homeostasis, promoting cellular zinc accumulation while decreasing cellular iron [109]. PBT2 lacked any impact on metal ion homeostasis such as iron, copper, and zinc in subjects recruited in Phase II clinical trials.

PBT2, in completed Phase II clinical trials for the treatment of neurodegenerative diseases such as Huntington’s and Alzheimer’s diseases, has been shown to be safe and well tolerated [110,111]. De Oliveira et al. showed that PBT2 + zinc was able to resensitize *E. coli*, *K. pneumoniae*, *P. aeruginosa*, and *A. baumannii* to both old polymyxins and F287 in a dose-dependent manner, in vitro [109]. Resistant mutant selection could not be achieved against a PBT2 plus F287 combination in the *mcr-1* gene carrying polymyxin-resistant *E. coli* and/or *K. pneumoniae* harboring an *mgrB* mutation in serial passaging experiments [109]. In a sepsis model of immunocompetent mice, both PBT2 plus colistin and PBT2 plus F287 were found highly effective against genetically engineered mgrB mutation carrying highly virulent *K. pneumoniae* strain [109]. Consistently, in a murine wound model of infection utilizing the polymyxin-resistant *K. pneumoniae* strain, a polymyxin plus PBT2 combination significantly reduced the bacterial load at the infection side [109]. Intriguingly, PBT2 plus zinc restored colistin activity in intrinsically colistin-resistant Gram-positive bacteria, including Group A *Streptococcus*, methicillin-resistant *Staphylococcus aureus*, and vancomycin-resistant *Enterococcus faecium* [112]. In a murine wound infection model targeting a Group A *Streptococcus* strain, the PBT2 plus colistin combination had a bactericidal effect [112]. Consequently, the utility of next-generation polymyxins plus PBT2 for the treatment of resistant Gram-positive bacterial infections can be investigated in future studies.

Well-designed PK/PD or toxicity studies have not yet been conducted for PBT2 plus polymyxins. In addition, the clinical utility of PBT2 + polymyxins, particularly the next-generation polymyxins, cannot be appreciated until human trials assessing the efficacy and safety of these combinations are performed.

## 6. Conclusions

Given the scarcity of alternative agents for CRAB and metallo-β-lactamase-producing GNB infections, it is indisputable that next-generation polymyxins will have significant potential in this area for improving therapy beyond the current standard of care. There are several polymyxin derivatives whose development is still in its infancy and have not yet reached Phase I clinical trials. Among polymyxin derivatives reached and/or completed Phase I trials, SPR206, MRX-8, and QPX9003 have direct antibacterial activity against GNB. Although SPR206 and MRX-8 did not confer any benefit over old polymyxins against polymyxin-resistant strains, QPX9003 had a significant bactericidal effect against polymyxin-resistant GNB strains in neutropenic mice pneumonia models. Lastly, PBT2 may play a crucial role when combined with direct-acting new-generation polymyxins against infections caused by polymyxin-resistant GNB. Such combinations could even be employed in the treatment of resistant Gram-positive bacterial infections caused by vancomycin-resistant *E. faecium* and/or methicillin-resistant *S. aureus*. This review article provides the most recent data and state-of-art status of the most promising polymyxin analogues and PBT2. Nevertheless, it is evident that there is a need for systematic reviews and meta-analyses that include randomized controlled clinical trials in which new polymyxins are tested, as well as studies investigating the anti-biofilm activities of new polymyxin molecules.

## Figures and Tables

**Table 1:** The structure of colistin, polymyxin B, and new polymyxin derivatives.

Compound	R1	R2	R3	R4	R5	R6	R7	R8	R9	R10	N-Terminus
Colistin	-Dab	-Thr	-Dab	-Dab	-Dab	-DLeu	-Leu	-Dab	-Dab	-Thr	Methyloctanoyl/methylheptanoyl
PMB	-Dab	-Thr	-Dab	-Dab	-Dab	-DPhe	-Leu	-Dab	-Dab	-Thr	Methyloctanoyl/methylheptanoyl
SPR206	-	-Thr	-Dab	-Dab	-Dab	-DPhe	-Leu	-Dab	-Dab	-Thr	(3S)-4-amino-3-(3-cholorophenyl)butanoyl
MRX-8	-Dab	-Thr	-Dab	-Dab	-Dab	-DPhe	-Leu	-Dab	-Dab	-Thr	3-(2,2-Dimethyl-butanoyloxy)-propanoyl (ester bond)
SPR741	-	-Thr	-DSer	-Dab	-Dab	-DPhe	-Leu	-Dab	-Dab	-Thr	Acetyl
QPX9003	-Dab	-Thr	-Dap	-Dab	-Dab	-DLeu	-Abu	-Dab	-Dab	-Thr	2,4 Dicholorobenzoyl

PMB, polymyxin B; Dab, diaminobutyric acid; Dap, diaminopropionic acid; Abu, L-2-aminobutyric acid; Ser, serine; Leu, leucine; Phe, phenylalanine; Thr, threonine.

## Data Availability

Not applicable.
